# Why public health needs evidence-informed guidelines for social media use

**DOI:** 10.3389/ijph.2026.1610087

**Published:** 2026-07-09

**Authors:** Jean-Philippe Chaput, Gary S. Goldfield

**Affiliations:** Children’s Hospital of Eastern Ontario Research Institute, Ottawa, ON, Canada

**Keywords:** adolescents, mental health, public health, sleep health, social media

## Introduction

Social media use has become nearly universal among adolescents and increasingly common across all age groups. Recent estimates suggest that adolescents spend several hours per day on social media platforms, often extending into late evening and nighttime hours [1]. Concurrently, growing evidence links excessive or problematic social media use with poor sleep, depressive symptoms, anxiety, body dissatisfaction, cyberbullying, sedentary behaviour, and impaired cognition and academic functioning [2–5]. Despite these concerns, evidence-informed recommendations for healthy social media use remain largely absent. Unlike tobacco, alcohol, nutrition, sleep, or physical activity, there are no widely accepted population-level guidelines to help individuals, families, schools, clinicians, or policymakers navigate digital exposure in a health-promoting manner.

The field of preventive medicine has historically focused on modifiable behavioural exposures that are widespread, begin early in life, and influence long-term health trajectories. Social media now meets many of these criteria. Exposure begins during childhood or adolescence, typically occurs daily for prolonged periods, and is shaped by commercial environments specifically designed to maximize user engagement. Importantly, social media use may influence health through several interconnected pathways, including sleep disruption, emotional arousal, social comparison, reduced physical activity, compulsive engagement patterns, and adverse associations with brain structure and function, particularly in the regions involved in motivation and reward processing as well as the prefrontal cortex, which governs attention and executive functioning [2–5]. Emerging evidence also suggests dose-response associations, where higher exposure or more problematic patterns of use are associated with poorer mental and physical health outcomes [2–5].

Social media is not inherently harmful and may provide important benefits, including opportunities for social connection, self-expression, peer support, health information seeking, and dissemination of public health messages. Social media platforms are increasingly used by public health organizations, healthcare professionals, and researchers to promote health literacy, communicate evidence-based information, and engage hard-to-reach populations. The challenge for public health is therefore not to discourage social media use altogether, but rather to identify patterns of use that maximize benefits while minimizing potential harms.

## Sleep as a key mechanistic pathway

Sleep health may represent one of the most immediate and biologically plausible pathways linking social media use with adverse health outcomes. Evening and nighttime social media use can delay bedtimes, increase cognitive and emotional arousal, expose users to stimulating content, and contribute to frequent nighttime awakenings caused by notifications [6]. Exposure to blue light emitted from screens may also delay circadian timing and reduce melatonin secretion. In adolescents, insufficient and irregular sleep are associated with poorer mental health, obesity, cardiometabolic risk factors, reduced academic performance, and increased healthcare utilization [7, 8]. Importantly, adolescence is also a developmental period characterized by heightened vulnerability to social evaluation and emotional reward processing, potentially amplifying the effects of social media engagement. Evidence supporting a causal relationship between social media use and adverse mental health outcomes is also emerging, including meta-analyses showing that reducing social media use may confer psychological benefits [9].

## Toward evidence-informed guidelines

Current approaches to social media and health remain fragmented and inconsistent. Schools are increasingly implementing smartphone restrictions or social media bans during instructional hours, but these policies vary substantially and are rarely grounded in standardized evidence-based recommendations. Parents often receive conflicting advice regarding appropriate levels of screen exposure, while clinicians lack practical guidance for counseling families about healthy digital behaviours. Existing recommendations frequently focus on total recreational screen time, despite evidence suggesting that different digital activities may carry different health implications [10]. For example, passive scrolling and nighttime social media engagement may have different effects compared with educational activities, active communication, or creative digital engagement. Recent studies published in the International Journal of Public Health further support the importance of considering the nature of social media engagement rather than exposure alone. Aryal et al. [11] reported that greater focus on self-presentation and social comparison on social media was associated with poorer mental wellbeing among early adolescents. Similarly, Boniel-Nissim et al. [12] found that different categories of social media use were differentially associated with body image concerns among adolescents across 42 countries. Together, these findings suggest that the quality, purpose, and context of social media engagement may be as important as duration of use when evaluating potential health effects and developing future public health recommendations.

Furthermore, social media exposure and its potential health consequences are unlikely to affect all populations equally. Adolescents, individuals with pre-existing mental health vulnerabilities, and socioeconomically disadvantaged groups may be particularly susceptible to problematic patterns of use and harmful online experiences [2–5]. Social media may also amplify existing health inequities through differential exposure to cyberbullying, misinformation, targeted advertising, and unhealthy social comparison. Future recommendations should therefore incorporate equity considerations and avoid approaches that disproportionately place responsibility on families with fewer social or economic resources.

The absence of fully developed evidence-informed public health guidelines does not imply that policy action should be delayed. Emerging evidence suggests that children and younger adolescents may represent a developmentally vulnerable population with increased susceptibility to potential harms associated with social media use, including effects on sleep, mental health, emotional regulation, and behavioural functioning [2–5]. In response, some jurisdictions such as Australia have adopted age-based restrictions for social media access among youth under 16 years, and similar discussions are ongoing in other countries. A balanced policy approach should therefore move toward a nuanced framework that combines evidence-informed policy measures with harm reduction and healthy digital behaviour strategies. Similar to modern approaches for physical activity and sleep, future social media recommendations could emphasize behavioural principles in addition to age-appropriate safeguards. Potential recommendations may include promoting device-free sleep environments, discouraging nighttime social media use, encouraging intentional rather than passive engagement, turning off notifications, establishing phone-free times and zones within the home, and supporting the development of non-screen, health-promoting activities such as physical activity, sport participation, hobbies, arts, and creative pursuits that may help youth adopt a more balanced and moderate approach to digital media use. Recommendations could also include integrating digital literacy into school curricula and supporting parents in establishing healthy digital routines at home. Clinicians may also benefit from practical screening tools and brief counseling approaches to help identify problematic social media use, particularly when evaluating sleep problems, emotional distress, or behavioural difficulties among adolescents.

## Beyond individual responsibility: structural and commercial determinants

A public health framework for social media use should also recognize broader structural and commercial determinants of health. Many platforms are intentionally designed to maximize engagement through personalized algorithms, infinite scrolling, social reinforcement, and frequent notifications. These features may disproportionately affect adolescents and other vulnerable populations. Consequently, responsibility for healthier digital environments should not rest solely on individuals or families. Policymakers, educators, public health agencies, and technology companies all have important roles in developing healthier digital ecosystems that prioritize user wellbeing alongside engagement metrics.

Researchers, clinicians, and policymakers can also contribute by strengthening surveillance and research efforts. Current surveillance systems often rely on simplistic self-reported measures of “screen time” that fail to capture the complexity of modern digital behaviours. Future surveillance efforts should distinguish between types of digital engagement, timing of use, emotional experiences, and patterns of problematic or compulsive use. Longitudinal studies and more sophisticated digital behaviour assessment methods may further improve understanding of dose-response associations and vulnerable developmental periods.

Societal responses to emerging behavioural exposures have historically evolved slowly. Tobacco use, unhealthy food environments, and sedentary lifestyles were all initially framed primarily as matters of individual responsibility before broader environmental and commercial determinants were recognized. Although social media differs in important ways and can provide important social, educational, and public health benefits, including opportunities for health promotion, health communication, and access to supportive online communities, the rapid rise of algorithm-driven digital environments raises concerns regarding persuasive design, prolonged exposure, and population-level behavioural influence. Growing legal and regulatory scrutiny of major technology companies over allegedly addictive platform features and insufficient protections for youth further supports the need to consider social media within a commercial determinants of health framework, particularly as some platform features may exploit developmental vulnerabilities in children and adolescents. There is now an opportunity to proactively develop balanced, evidence-informed guidelines before harmful patterns become further normalized across generations.

Over time, evidence-informed recommendations have been developed for many behavioural exposures that were once considered matters of personal choice. The absence of evidence-informed guidelines amidst widespread and prolonged social media exposure now represents an important gap in modern preventive medicine. Developing balanced, evidence-informed guidelines for healthy social media use may represent an important step toward supporting sleep health, mental wellbeing, and healthier digital environments across the lifespan. Future guidelines should aim to preserve the potential social, educational, and health-promoting benefits of social media while reducing exposure to features and patterns of use that may adversely affect health and wellbeing.
